# *Helicobacter pylori* vaccines: from bench to bedside—progress, challenges, and future directions

**DOI:** 10.3389/fimmu.2026.1918975

**Published:** 2026-09-14

**Authors:** Shue Wang, Yong Yang, Chunyan Ma, Le Guo

**Affiliations:** 1 Ningxia Key Laboratory of Clinical and Pathogenic Microbiology, General Hospital of Ningxia Medical University (The First Clinical Medical College of Ningxia Medical University), Yinchuan, China; 2 Basic Medical College, Ningxia Medical University, Yinchuan, China; 3 Ningxia Key Laboratory of Craniocerebral Diseases, Ningxia Medical University, Yinchuan, China; 4 Tumor Hospital, General Hospital of Ningxia Medical University, Yinchuan, China

**Keywords:** delivery system, *H. pylori*, mucosal adjuvant, mucosal immunity, vaccine

## Abstract

*Helicobacter pylori* (*H. pylori*) is a major human pathogen that colonizes the gastric mucosa and is a principal etiological agent of gastritis, peptic ulcer disease, and gastric cancer. Although antibiotic-based eradication regimens are available, their cost and the poor prognosis of established gastric cancer make vaccination an economically attractive and potentially more durable alternative. This narrative review synthesizes the literature on *H. pylori* pathogenesis, host protective immunity, and vaccine development, with particular attention to the human clinical trials reported since 1999 and with emphasis on preclinical work published since 2020. Rather than cataloguing candidate antigens, we organize the field into three interdependent tiers—classical approaches, emerging technologies, and the enabling adjuvant and delivery platforms on which both depend—and use this framework to examine why preclinical success has repeatedly failed to translate into clinical protection, and to identify the principal challenges now facing human *H. pylori* vaccine development. We conclude that the principal bottleneck is no longer antigen discovery but antigen delivery and immune programming at the gastric mucosa.

## 1 Introduction

*Helicobacter pylori* (*H. pylori*) is uniquely adapted to the harsh environment of the human stomach, where it colonizes the gastric mucosa and can persist for decades unless eradicated by antimicrobial therapy (1). The organism infects more than half of the world’s population and is a major etiological agent of gastrointestinal disease (2), including chronic gastritis, peptic ulcer disease, and gastric adenocarcinoma (3). In 1994, the International Agency for Research on Cancer (IARC), an agency of the World Health Organization (WHO), classified *H. pylori* as a Group 1 carcinogen, confirming it as a definitive cause of cancer in humans (4). The clinical value of controlling the infection is well established: an 11-year follow-up study (2011–2022) demonstrated that eradication of *H. pylori* reduced the incidence of gastric cancer by 19% (5).

Antibiotic-based eradication is nevertheless an increasingly fragile foundation for population-level control. *H. pylori* readily acquires resistance to multiple antibiotics (6), and approximately 10% of strains worldwide are resistant to metronidazole and clarithromycin (7), progressively undermining the efficacy of standard regimens. Successful eradication, moreover, does not confer lasting protective immunity, so cured individuals remain fully susceptible to reinfection (8); annual reinfection rates reach 15-30% in some regions (9). A further obstacle is that infection is clinically silent in the great majority of carriers. Only about 10% of infected individuals ever develop overt disease such as peptic ulceration or gastric cancer; the remaining 90% carry the organism without symptoms that would prompt them to seek medical attention. Because those who will progress cannot be identified in advance, symptom-driven case finding is inadequate, and effective control requires active screening of entire populations followed by treatment of all who test positive. The combined diagnostic and pharmaceutical cost of such a strategy is prohibitive for many health systems.

Given the high global prevalence of infection, the rapid emergence of drug-resistant strains, and the substantial cost of test-and-treat programs, vaccination represents a rational and potentially transformative strategy for controlling this pathogen (7, 10). Yet, despite more than three decades of research, no *H. pylori* vaccine has been licensed. Designing a protective *H. pylori* vaccine is not just about choosing the right antigens. We also need to understand why the host fails to clear the bacterium naturally, and how a vaccine might help at both the mucosal and systemic levels. In this review, we revisit the pathogen’s evasion strategies and its interplay with the host, before surveying the evolving vaccine landscape, from whole-cell and subunit approaches to multi-epitope designs, nucleic acid platforms, and artificial intelligence (AI)-driven target discovery. We also examine recent advances in adjuvants and delivery systems, and conclude by outlining the key obstacles to clinical translation.

We have drawn on recent excellent reviews but have not tried to repeat them. This review is organized around a few key distinctions. **Table 1** outlines a three-tier framework. It sets classical approaches apart from newer antigen-design technologies, and both from the adjuvants and delivery systems they require. This organization makes one point clear. The field’s bottleneck has shifted. The main challenge is not finding new antigens. It is delivering them to the gastric mucosa. We also tackle a persistent paradox, namely the presence of a strong yet non-protective Th1 response during natural infection versus its apparent necessity in vaccination, and we discuss what this means for adjuvant choice and immune endpoints (section 3.2). Finally, we distinguish explicitly between computational predictions and experimental validation-particularly for self amplifying RNA (saRNA), circular RNA (circRNA), and AI-designed antigens to avoid overinterpreting *in silico* results.

**Table 1:** A three-tier framework for *H. pylori* vaccine technologies.

Category	Representative technologies	Primary function	Current development stage for *H. pylori*
Classical approaches	Whole-cell vaccines, Subunit vaccine	Conventional empirical antigen selection using biochemical and immunological assays.	Clinical trials with suboptimal protective efficacy.
Emerging technologies	Immunoinformatics-based multi-epitope vaccines, DNA/mRNA vaccines, AI-driven target discovery, validation, and optimization.	Rational antigen design through *in silico* prediction.	Preclinical to early-phase clinical exploration.
Enabling technologies	Delivery systems, Advanced adjuvant	Potentiate and direct immune responses; protect and deliver antigens to mucosal surfaces.	Preclinical validation in animal models.

## 2 Pathogenesis and immune evasion of *H. pylori*

### 2.1 Pathogenic mechanism of *H. pylori*

The pathogenicity of *H. pylori* is a multi-step, multifactorial process that proceeds from survival in the gastric lumen, through penetration of the mucus layer and adhesion to the epithelium, to the delivery of virulence factors and the establishment of persistent inflammatory damage (11). The first requirement is survival in gastric acid: *H. pylori* secretes highly active urease that hydrolyzes host urea to ammonia (NH_3_) and carbon dioxide (CO_2_), generating a PH-neutral “ammonia cloud” around the bacterium that buffers gastric acid and permits transient survival in the lumen (12). Next, the bacterium needs to escape from the lumen into the protected mucus niche, and transmigration across the gastric luminal barrier represents the initial and indispensable step in pathogenesis. The helical morphology and unipolar flagella of *H. pylori* allow it to move rapidly through the viscous gastric mucus layer and to reach the surface of the gastric epithelium. Whereas urease supports colonization indirectly by neutralizing acid, flagellar motility serves as a direct mechanical countermeasure against gastric motility-mediated clearance (13). Finally, stable attachment is required. Outer membrane adhesins, notably the blood-group antigen-binding adhesin (BabA) and sialic acid-binding adhesin (SabA), bind the Lewis b blood group antigen and sialylated Lewis x antigen, respectively, on the gastric epithelial surface, anchoring the bacterium against clearance by gastric peristalsis and emptying (14, 15). *H. pylori* outer membrane protein Q (HopQ) engages host carcinoembryonic antigen-related cell adhesion molecules (CEACAMs); this interaction not only strengthens adhesion but also provides the gateway for the subsequent translocation of the cytotoxin-associated gene A protein (CagA) into the cell (16).

Following successful colonization, *H. pylori* directly disrupts host cellular architecture and perturbs key signaling pathways through its core virulence determinants. Central to these pathogenic mechanisms are the type IV secretion system (T4SS), CagA, and vacuolating cytotoxin A (VacA) (13, 16). Strains possessing the CagA gene assemble a T4SS that functions as a “molecular syringe” to precisely inject the CagA protein into gastric epithelial cells with the assistance of the HopQ adhesin (17). Once inside the cell, CagA abnormally activates carcinogenic signaling pathways, induces epithelial-mesenchymal transition (EMT), and confers invasive properties to normal epithelial cells (18, 19). VacA is another major virulence factor that forms vesicular channels within cells, disrupts membrane structures, and ultimately leads to characteristic “vacuole” formation (20). VacA toxicity is enhanced by the ammonia produced by urease, resulting in synergistic destructive effects (16). Concurrently, VacA targets the mitochondrial inner membrane via N-terminal anchoring and oligomerization of the p34 subunit. This process forms a hexameric anion channel that disrupts ionic homeostasis and membrane potential, thereby triggering bioenergetic collapse and subsequent apoptosis (21). VacA can also inhibit T-cell and B-cell activation, helping *H. pylori* evade immune attack (13). The neutrophil-activating protein (NAP) is a key virulence determinant with a dual role: it not only exacerbates​ mucosal inflammation through immune cell activation and pro-inflammatory cytokine release but also confers bacterial resistance to oxidative stress by chelating iron​ and binding DNA, thereby ensuring persistent colonization (22, 23).

In summary, the pathogenic strategy of *H. pylori* reflects the long co-evolutionary relationship between the bacterium and its host. The organism first neutralizes gastric acid through urease activity, penetrates the mucus layer by flagellar motility, and anchors itself to the epithelium via outer membrane protein-mediated adhesion; it then deploys the T4SS, CagA, VacA, and NAP to damage host cells, subverts intracellular signaling, and perpetuates mucosal inflammation. Chronic inflammation driven by these processes progressively remodels the gastric mucosa and promotes malignant transformation (**Figure 1**). Together, these mechanisms account for its classification by the IARC as a Group 1 carcinogen (24).

**Figure 1:**
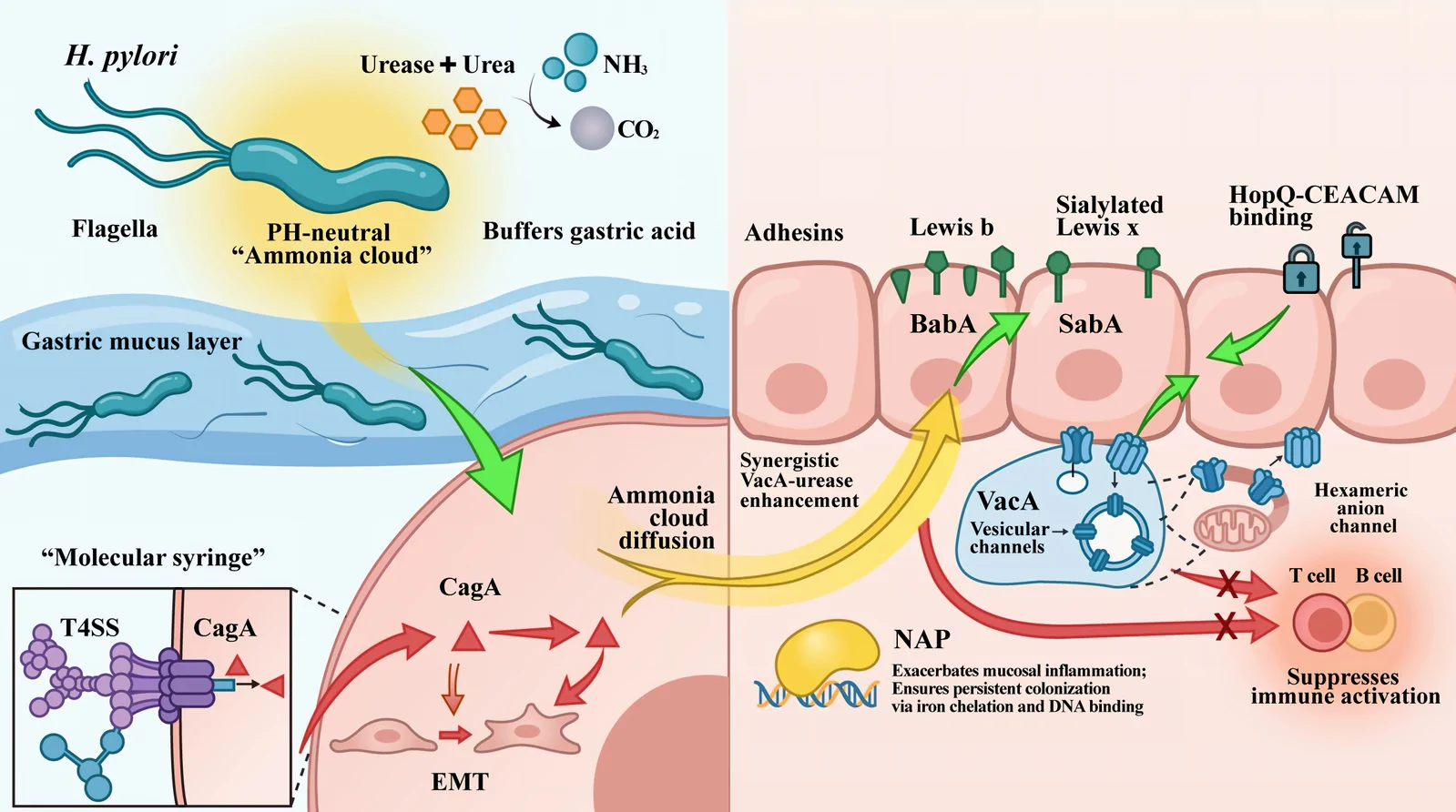
Schematic diagram of the pathogenic mechanism of *H. pylori* virulence factors. *H. pylori* pathogenesis proceeds through gastric acid survival, mucus penetration, epithelial adhesion, virulence factor delivery, and persistent inflammation. Urease produces an “ammonia cloud” that neutralizes gastric acid, while flagella propel the bacterium through the mucus. Adhesins (BabA, SabA, HopQ) anchor the bacterium to the epithelium. The T4SS injects CagA into host cells as a “molecular syringe”, thereby inducing EMT. VacA forms hexameric anion channels and induces vacuolation, causing apoptosis, and its toxicity is enhanced by urease-derived ammonia. VacA also suppresses T cell and B cell activation. NAP drives mucosal inflammation and ensures persistent colonization via iron chelation and DNA binding. Arrow color codes: green arrows indicate activation or colonization-promoting effects; red arrows indicate inhibition, damage, or pathological transformation; yellow arrows indicate synergistic enhancement. T4SS, type IV secretion system; CagA, cytotoxin-associated gene A; VacA, vacuolating cytotoxin A; NAP, neutrophil-activating protein; EMT, epithelial–mesenchymal transition. Created with Adobe Illustrator.

### 2.2 Immune evasion mechanism of *H. pylori*

Despite eliciting a robust host immune response, *H. pylori* achieves lifelong colonization through multilayered evasion strategies encompassing innate avoidance, adaptive regulation, and phenotypic variation (25). A primary mechanism is lipid A remodeling: dephosphorylation and acyl chain elongation (C16/C18) generate a tetra-acylated lipid A that is poorly recognized by the TLR4/MD-2 complex, suppressing pro-inflammatory cytokine release, such as tumor necrosis factor-alpha (TNF-α) (26, 27). The pathogen further employs molecular mimicry, expressing host Lewis antigens (Le^x^/Ley) to disguise itself as host tissue (26). Beyond passive camouflage, Le^+^ variants ligate DC-SIGN on dendritic cells, actively suppressing Th1 polarization and fostering an immunosuppressive niche (28). Concurrently, specific mutations in *H. pylori* flagellin (such as R89, L93) disrupt TLR5 binding, inhibiting NF-κB activation and permitting unimpeded mucosal motility (29). This is complemented by phase variation, wherein slipped-strand mispairing in O-antigen biosynthetic genes drives ON/OFF switching of Lewis antigens; this generates antigenic heterogeneity, allowing Le^−^ variants to evade anti-Le^+^ immunity and maintain strain diversity (26, 30, 31). Finally, *H. pylori* subverts immunity by expanding regulatory T cells (Tregs) and skewing cytokine profiles towards an anti-inflammatory state characterized by elevated interleukin-10 (IL-10) and reduced interleukin-12 (IL-12) (32).

Together, these mechanisms facilitate decades-long persistence, driving progression from chronic gastritis to gastric malignancy. Nevertheless, translating these mechanistic insights into effective immunotherapies has been hampered by the marked heterogeneity of Lewis antigen expression and the lack of reliable biomarkers for predicting vaccine efficacy in high-risk populations.

## 3 Host immune responses underlying vaccine-mediated protection

Advances in *H. pylori* vaccinology are predicated on deciphering the host immune response to guide antigen design. Infection elicits a biphasic immune response: while the innate arm​ is rapidly mobilized, it invariably fails to eradicate the pathogen. This is followed by adaptive immunity, engaging both humoral and cellular components. Despite this robust induction, the response is non-sterilizing, leading to chronic immune activation (33, 34). Consequently, vaccine research is strategically structured around two evaluative pillars: the induction of protective humoral immunity and the orchestration of effective cellular immunity.

### 3.1 The role of humoral immunity in *H. pylori* vaccine protection

Humoral immunity is a critical component of vaccine-induced protection against this pathogen (35). Humoral immunity is mediated by B cells, which, upon activation, differentiate into plasma cells and produce specific antibodies. These antibodies primarily target various *H. pylori* antigenic proteins, such as urease, heat shock protein, CagA, and VacA (**Table 2**) (1). Given that *H. pylori* is a mucosa-associated pathogen, it has been hypothesized that secretory IgA (sIgA) antibodies are critical for protective immunity. Early studies supported this, suggesting that sIgA contributes to protection against infection (49, 50). Furthermore, multiple studies have confirmed that inoculation with an *H. pylori* vaccine significantly increases anti-*H. pylori* IgA levels in saliva and gastric mucosa, as well as the percentage of sIgA-positive glands in the gastric mucosa. These increases are positively correlated with the vaccine’s protective efficacy (51). Additionally, serum-specific IgG levels serve as an important indicator of the systemic humoral immune response induced by vaccination. For instance, a study utilizing genetically engineered *Lactococcus lactis* to express and deliver the *H. pylori* Lpp20 antigen demonstrated that oral vaccination significantly elevated Lpp20-specific serum IgG levels in BALB/c mice (*P* < 0.05) (52), indicating a robust systemic humoral response. The predominant protective role of IgA has, however, been challenged by evidence from knockout mouse models (53–55). For example, vaccine-induced protection was shown to be equivalent in IgA-deficient knockout mice and immunized wild-type mice (55), suggesting that antibody-independent immune mechanisms can confer protection against *H. pylori*. This is further supported by the clinical observation that the prevalence of *H. pylori* infection does not differ significantly between IgA-deficient patients and individuals with normal IgA levels (56).

**Table 2:** Main characteristics of the humoral immune response associated with *H. pylori* infection.

Characteristics of the immune response	Primary categories of relevant antibodies	Key features	Changes in disease progression
Local immune response of gastric mucosa (36, 37).	IgA	Neutralize toxins and inhibit bacterial adhesion.	Decreased in cancerous tissue, elevated in surrounding tissue.
Systemic immune response (38, 39).	IgG	Complement activation, phagocytosis regulation, serological diagnostic markers.	Continues to rise with disease progression (such as gastric cancer).
Vacuolating Cytotoxin A (VacA) (40, 41).	IgG, IgA	Diagnostic marker for detecting *H. pylori* infection.	Positively correlated with the severity of inflammation, and associated with the risk of ulcers and gastric cancer.
Urease-specific reaction (42–44).	IgG, IgA	For the widespread screening of *H. pylori* infection and as a target for vaccine development.	Significantly elevated following vaccination, promoting bacterial clearance.
Cytotoxin-associated protein A (CagA) reaction (41, 45, 46).	IgG, IgA	Markers of disease severity, associated with infection by virulent strains.	Higher titers were observed in patients with peptic ulcers and gastric cancer.
Heat shock protein (47, 48).	IgG	Associated with MALT lymphoma, involved in B-cell proliferation.	Significantly elevated in patients with MALT lymphoma.

In summary, the humoral immune response elicited by *H. pylori* infection exhibits a dual role. On one hand, specific antibodies can neutralize bacterial toxins, inhibit bacterial adhesion to gastric epithelial cells, activate the complement system, and enhance phagocytosis, thereby partially limiting the infection (57, 58). On the other hand, the antibody response may also exacerbate inflammation, contribute to tissue damage, and potentially participate in carcinogenic processes (36). It is therefore proposed that neutralizing antibodies targeting key *H. pylori* virulence or colonization factors can disrupt the niche required for bacterial persistence, thereby potentially contributing to the clearance of the infection (59–62).

### 3.2 The role of cellular immunity in *H. pylori* vaccine protection

T-cell-mediated immunity has a paradoxical dual role in *H. pylori* infection, underpinning both pathogen clearance and immunopathology (63). The importance of CD4^+^ T helper (Th) cells in protection against *H. pylori* is well-established (55, 64, 65). These cells are conventionally categorized into Th1, Th2, and Th17 subsets on the basis of their cytokine secretion profiles. Natural infection robustly induces a Th-polarized response characterized by the production of interferon-gamma (IFN-γ), interleukin-2 (IL-2), and TNF-α (66). This polarization is driven by bacterial virulence factors: CagA, delivered into host cells by the T4SS, activates NOD1 and NF-κB signaling and promotes the production of IL-12 and interleukin-18 (IL-18), which in turn drive the differentiation of naive T cells into Th1 cells (67–69). Concurrently, *H. pylori* employs multiple strategies to promote the expansion of Tregs, and thereby suppress host immunity. VacA binds receptors on gastric epithelial cells and T cells to gain entry, promotes epithelial cell death, and inhibits T cell activation: in the murine gastric mucosa it also impairs dendritic cell maturation and antigen presentation by modulating E2F transcription factor signaling, favoring the differentiation of naive T cells into Tregs (68, 70). The *H. pylori* heat shock protein 60 (Hsp60) likewise induces Treg activation. Once activated, Tregs strongly suppress effector T cells, including Th1 cells, through inhibitory cytokines, such as IL-10 and transforming growth factor-beta (TGF-β), or through cell contact-dependent mechanisms, facilitating immune evasion and persistent colonization while impeding pathogen clearance (71, 72).

The contributions of the individual Th subsets have proved difficult to disentangle, and the resulting picture appears contradictory at first sight. Th2 responses, such as those driven by interleukin-4 (IL-4) and interleukin-5 (IL-5), have been implicated in vaccine-mediated protection (73, 74); adoptive transfer of *Helicobacte*r-specific T cells indicated that Th2 cells can mediate protective immunity, whereas Th1 responses were associated with disease pathogenesis (73). Th17 cells, which secrete interleukin-17A (IL-17A), interleukin-17F (IL-17F), and interleukin-22 (IL-22), drive neutrophil recruitment and inflammation and act in concert with Th1 responses to sustain chronic gastritis (75). Protective vaccination typically elicits a transient, mucosal Th1/Th17-dominated inflammatory response that resembles early gastritis but resolves upon bacterial clearance. This acute immunopathological response is mechanistically distinct from the chronic, Treg-skewed inflammation that characterizes natural infection and drives disease progression. Early work nevertheless regarded the Th1 responses as the principal protective mechanism against *H. pylori*, even though vaccine strategies directed solely at Th1 immunity failed to achieve satisfactory outcomes in clinical trials (76). A persistent puzzle in *H. pylori* vaccinology is that the same Th1 response that dominates natural, non-sterilizing infection has also been reported to be required for vaccine-induced protection.

This apparent contradiction is not as puzzling as it seems. It disappears once we take into account the quality and the anatomical location of the response, not just the T-cell lineage. Three considerations explain why the earlier Th1-centric model proved inadequate. One consideration is that the Th1 response elicited by natural infection is not absent but ineffective. It is mounted against an actively expanded Treg compartment within an IL-10-rich, IL-12-poor cytokine milieu (32, 71, 72), with the consequence that IFN-γ production correlates with the severity of gastritis and with mucosal immunopathology rather than with bacterial clearance. IFN-γ was accordingly adopted as a surrogate of protection when it is, more accurately, a surrogate of inflammation. Another is that Th1 read-outs in early vaccine studies were obtained predominantly from systemic compartments, such as splenocyte recall assays and serum cytokine measurements, whereas the effector events that limit colonization occur in the gastric lamina propria; a vaccine could therefore appear strongly Th1-polarizing while failing to establish the local effector program required at the site of infection. A third is that the effector axis that recruits neutrophils to the gastric mucosa and disrupts the colonization niche—the IL-17A-dependent Th17 response—was not measured at all in those studies, and its contribution was consequently attributed to the Th1 response with which it is co-induced. Advances in mucosal immunology have since established an indispensable role for Th17 cells and IL-17A in local gastric defense (77).

The current consensus is therefore not that Th1 immunity is dispensable, but that no single subset is sufficient. Effective anti-*H. pylori* immunity requires the coordinated engagement of both arms, with Th1 responses providing systemic effector support and macrophage activation and Th17 responses orchestrating the local, neutrophil-dependent mucosal defense that natural infection, restrained by Tregs, fails to mount (78–80). This interpretation is consistent with experimental studies reporting that protection is mediated collectively by a strong, mixed Th response rather than by any single polarized profile (81–84), and it accommodates the earlier Th2 transfer data without requiring them to be discarded: those experiments demonstrate that protection can be achieved by more than one effector route, not that a particular lineage is uniquely protective (**Figure 2**).

**Figure 2:**
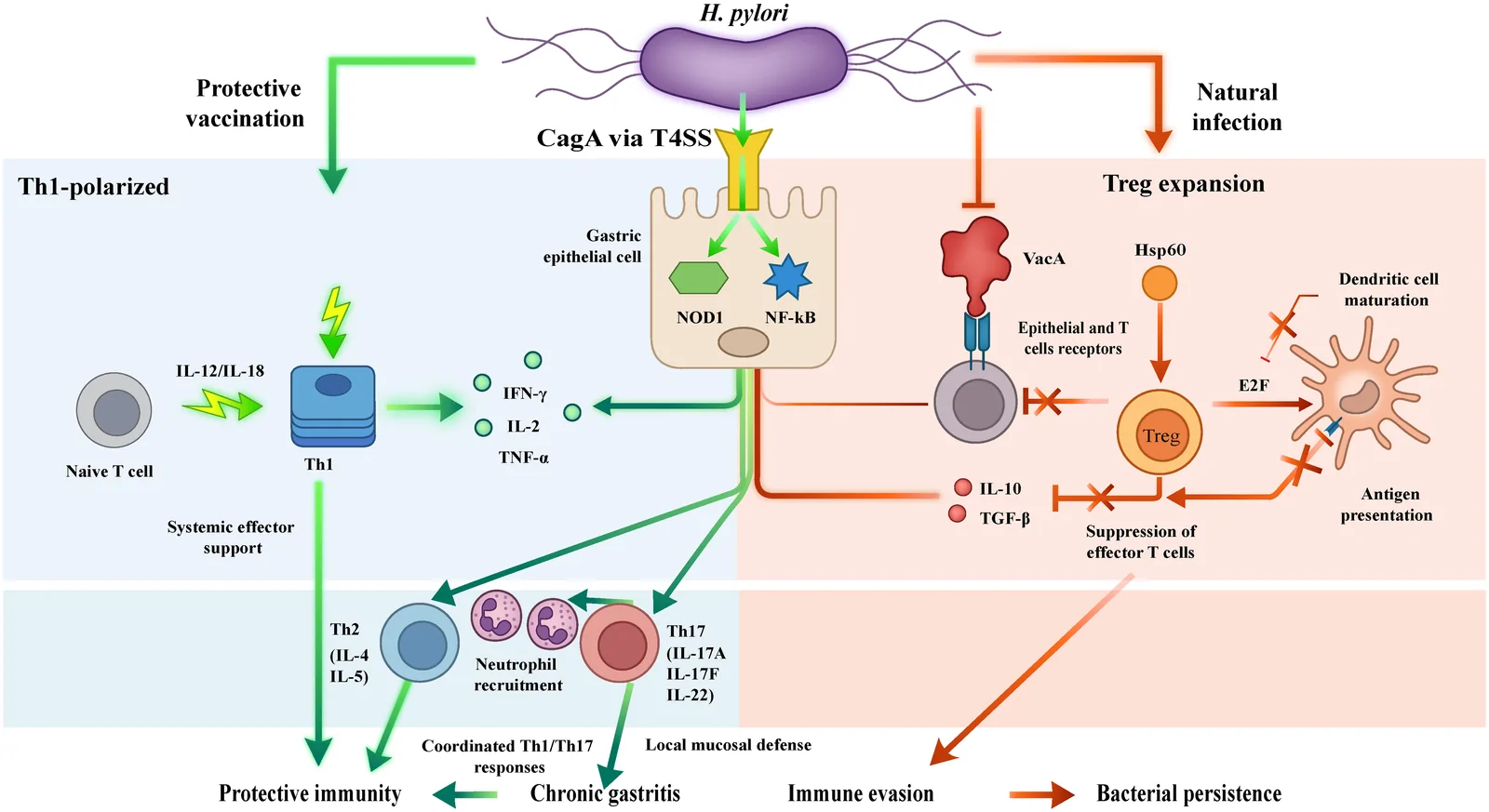
Contrasting immune responses between natural infection and vaccine-induced immunity: from Treg-mediated immune evasion to Th1/Th17 coordinated protection. Natural infection (red) drives Treg expansion via VacA and Hsp60, establishing a robust immunosuppressive niche that facilitates bacterial persistence. In contrast, protective vaccination (green) needs to overcome this suppression and integrate Th1, Th2, and Th17 effector arms. While CagA-induced Th1 cells provide systemic effector support and Th2 cells contribute to protective immunity, Th17 cells orchestrate local mucosal defense through neutrophil recruitment. This model underscores that effective vaccine-induced immunity should not rely on a single subset; rather, it demands the synergistic activation of Th1, Th2 and Th17 pathways within the gastric mucosa to dismantle Treg-mediated evasion and achieve protective immunity. Created with Adobe Illustrator.

These observations have several practical implications for vaccine design. To begin with, adjuvants and delivery systems should be selected for their capacity to co-induce Th1 and Th17 responses at the gastric mucosa, rather than for systemic IFN-γ induction alone; formulations that meet this criterion, including the double mutant heat-labile toxin (dmLT), combined Toll-like receptor 3 (TLR3) and Toll-like receptor 9 (TLR9) agonists, and cytokine-armed vesicle platforms delivering IL-17A and IFN-γ, are discussed in sections 5.3 and 6.2. Next, immunological endpoints in preclinical and early clinical studies should report IL-17A and mucosal secretory IgA alongside IFN-γ and serum IgG, together with an assessment of the Treg compartment, because a vaccine that raises effector cytokines without relieving regulatory suppression is unlikely to translate. In addition, since the margin between protective Th17 immunity and Th17-driven immunopathology is narrow, dose and adjuvant optimization should be evaluated against gastritis severity as well as bacterial load. A few points are worth stressing here. For human *H. pylori* infection, we still lack a validated correlate of protection; indeed, most of what we think we know about how Th1 and Th17 cells contribute to gastric mucosal immunity still comes from mouse studies. With these gaps still unresolved, building a vaccine strategy around a single assumed mechanism seems premature.

The relationship between these T-cell subsets is complicated, and it continues to obscure the real basis of protective immunity. Going forward, researchers should connect specific immune profiles, particularly T-cell polarization and cytokine patterns, more directly to how the disease progresses. Getting those connections right will be important, not just for predicting clinical outcomes, but also for designing more rational immunotherapies.

## 4 Classical approaches: lessons from clinical trials

### 4.1 Whole-cell vaccines

Whole-cell vaccines represent the oldest approach in *H. pylori* vaccine research, using intact but inactivated bacteria as the immunogen to prime the immune system against the organism (85). Compared with the more widely studied subunit vaccines, which employ only a small number of defined bacterial proteins, whole-cell preparations retain the complete antigenic repertoire of *H. pylori*. In principle, this broader repertoire should elicit stronger protection and be applicable to both prevention and treatment (18, 86). In practice, however, this same completeness has become the principal obstacle to clinical application. The crude antigen composition of whole bacteria is extremely complex, and it is difficult to eliminate entirely the associated toxicity, carcinogenic potential, and risk of autoimmune cross-reactivity (18). Trace amounts of toxins such as VacA and other harmful components may persist in bacterial extracts, and this residual risk is not fully abolished by inactivation (87). Because of these safety concerns, there is broad agreement that the direct use of crude whole-cell antigens for human vaccination faces significant hurdles.

In animal models, whole-cell vaccines have shown encouraging prophylactic and therapeutic potential. Early preclinical work established that inactivated whole-cell preparations administered alone were insufficient to elicit robust immunity; co-administration with a potent mucosal adjuvant, such as cholera toxin (CT) or *Escherichia coli* heat-labile enterotoxin (LT), was indispensable for inducing protective responses in mice (85). In a pivotal 2016 study, therapeutic immunization of *H. pylori*-infected mice with a heat-inactivated whole-cell vaccine formulated with aluminum phosphate and delivered by a systemic route (intramuscular or subcutaneous) elicited high-titer antigen-specific IgG antibodies and markedly reduced gastric bacterial loads, confirming the therapeutic potential of whole-cell vaccines *in vivo* (88). These results were nevertheless obtained in rodents whose gastric immunology differs substantially from that of humans, and they were not reproduced in the clinic.

The most definitive clinical investigation of this vaccine type remains a Phase I trial reported in 2001 (89). The study demonstrated that the whole-cell vaccine was capable of eliciting an immune response, but only at a high antigenic dose: significant immunological reactivity was observed only in subjects receiving the highest dose (2.5 ×10¹^0^ bacterial cells) with adjuvant. This high-dose regimen produced more pronounced responses in *H. pylori*-uninfected volunteers, as evidenced by significant lymphocyte proliferation in 5 of 10 individuals and *H. pylori*-specific IFN-γ production in 7 of 10 individuals, indicating cellular immune activation. The magnitude of this activation nevertheless remained constrained: only modest elevations in antigen-specific serum IgA and IgG titers were detected, predominantly in pre-infected subjects, and the frequency of induced antibody-secreting cells remained minimal. Critically, the primary endpoint of therapeutic eradication was not achieved. The safety profile also raised non-negligible concerns, with diarrhea in 6 of 18 recipients (33.3%) of the highest-dose regimen, low-grade fever in 5 cases, and vomiting in 2 cases. Most decisively, no evidence of *H. pylori* clearance was documented in any vaccinated, infected participant.

Taken together, this trial established three points that have shaped subsequent work. Immunogenicity in humans required an antigen dose at the upper limit of what can be manufactured and tolerated; the responses obtained were cellular rather than humoral in character; and measurable immune activation did not translate into any reduction in bacterial burden. Whole-cell preparations have therefore been abandoned as a route to therapeutic eradication, and the field has shifted toward defined antigens that can be delivered at lower doses with better-characterized adjuvants.

### 4.2 Subunit vaccines

*H. pylori* subunit vaccines represent the second generation of vaccine design. Rather than whole-cell vaccines, they employ genetic engineering and related technologies to obtain defined protective antigens, such as the *H. pylori* urease B subunit (UreB) and heat-shock protein A (HspA), that are capable of stimulating an immune response (90). Because subunit vaccines contain no bacterial genetic material, vaccination carries no risk of infection, which is their principal advantage over traditional whole-cell inactivated or attenuated preparations. They are also highly amenable to design, allowing several protective antigens to be combined into multivalent formulations for broader coverage (18). Their immunogenicity, however, is generally inferior to that of whole-bacterial vaccines precisely because they present only a fraction of the pathogen, and they must be combined with a potent adjuvant to elicit sufficient protection (91). Because *H. pylori* colonizes the gastric mucosal surface, an ideal vaccine must simultaneously stimulate systemic humoral immunity, local mucosal immunity mediated by secretory IgA, and cellular immunity (92). The immune mechanisms engaged by subunit vaccines are correspondingly complex, and the efficient induction of mucosal immunity has remained the central technical challenge in their development.

Early preclinical investigations explored a range of candidate antigens, including the urease A subunit (UreA), UreB and NAP. In one representative study, oral immunization with recombinant *H. pylori* antigens (NAP, UreA, and UreB) formulated with the novel adjuvant dmLT produced a significant reduction in gastric bacterial colonization at 6 weeks after challenge, compared with unimmunized controls, and outperformed whole-cell vaccines, indicating the considerable prophylactic potential of multicomponent subunit formulations (93).Whereas whole-cell vaccines proved ineffective for therapeutic intervention, subunit vaccines have achieved a landmark success in prevention. The most notable accomplishment to date is an oral recombinant subunit vaccine that completed a Phase III clinical trial. In a randomized, double-blind, placebo-controlled trial in 4,464 *H. pylori*-naive children aged 6–15 years, the vaccine conferred a protective efficacy of 71.8% (95% CI 48.2–85.6) during the first year after immunization, which was sustained at approximately 65% (95% CI 46.4–77.7) over a 3-year follow-up period (94). This prophylactic success stands in marked contrast to the obstacles encountered in therapeutic trials aimed at eliminating established infection. In an early study of *H. pylori*-infected adults, oral immunization with recombinant urease adjuvanted with LT produced disappointing outcomes: no dose group achieved bacterial eradication, and the adjuvant was associated with prominent adverse events, including diarrhea in 16 of 24 participants (95). The contrast between these two outcomes is arguably the most informative observation in the human data set. A defined subunit antigen delivered orally with an engineered mucosal adjuvant can prevent the acquisition of infection in naive children, whereas no vaccine of this class has eradicated an established infection in adults.

Prevention and eradication are therefore viewed as points on a single continuum, yet they represent distinct immunological challenges. While the current focus remains on prophylaxis, reflecting this immunological asymmetry, it does not preclude therapeutic ambition. This distinction drives contemporary research toward refined antigen design and safer mucosal adjuvants.

### 4.3 Outcomes of human clinical trials and the barriers they reveal

A total of twelve human clinical trials of vaccines against *H. pylori* infection have been reported (**Table 3**). Their outcomes highlight several barriers that future vaccine candidates will need to address.

**Table 3:** Human clinical trials of *H. pylori* vaccines.

Author	Type	Antigen	Adjuvant or carrier	Route	Phase	Immune effect
Kotloff et al (89).	Therapeutic	*H. pylori* whole-cell vaccine	LT_R192G_	Oral	I	Anti-*H. pylori* serum and mucosal IgA and IgG increased. Diarrhea, low-grade fever, and vomiting were observed. There was no evidence that vaccination eradicated *H. pylori* in infected volunteers.
Michetti et al (95).	Therapeutic	Urease	LT	Oral	I	Anti-*H. pylori* serum IgA increased. Immunization induced a significant decrease in gastric *H. pylori* density. Diarrhea was noted in 16 of 24 of the volunteers.
Banerjee et al (96).	NA	Urease	LT	Oral	I	Anti-*H. pylori* serum IgA and IgG increased.
Sougioultzis et al (42).	NA	Urease	LT	Rectal	I	Anti-*H. pylori* serum IgA and IgG increased in <20% of individuals.
DiPetrillo et al (97).	NA	Urease	*Salmonella enterica* serovar Typhi Ty1033	Oral	I	None of three had detectable humoral or mucosal immune responses to urease.
Angelakopoulos et al. (98).	NA	Urease	*Salmonella enterica* serovar Typhimurium	Oral	I	Anti-*H. pylori* PBMC IgG ASCs in 50% of individuals increased. 2 of 6 volunteers had fever.
Bumann et al (99).	NA	Urease	*Salmonella enterica* serovar Typhi Ty21a	Oral	I	Weak immune responses were induced in *H. pylori*-negative individuals.
Metzger et al (100).	Prophylactic	Urease	*Salmonella enterica* serovar Typhi Ty21a	Oral	I	After vaccination, two volunteers produced an activated CD4 ^+^ cell response to natural urease. Two volunteers (No.13,14) developed vomiting symptoms 6 hours after the first dose of vaccine.
Aebischer et al (101).	Prophylactic	Urease or HP0231	*Salmonell*a *enterica* serovar Typhi Ty21a	Oral	II	*H. pylori*-specific Th cells were detected in 9 of these 13, but only in 6 of 45 breath test-positive volunteers. Vaccines did not show satisfactory protection.
Malfertheiner et al. (102).	NA	VacA, CagA, NAP	Aluminum hydroxide	IM	I	Anti-*H. pylori* serum IgG increased in 86% of individuals. Strong humoral and cellular memory response upon repeated vaccination after 18 months.
Malfertheiner et al. (103).	Prophylactic	VacA, CagA, NAP	Aluminum hydroxide	IM	I/II	The vaccine did not confer additional protection against *H. pylori* infection after challenge with a CagA-positive strain.
Zeng et al. (94).	Prophylactic	UreB	LTA2B	Oral	III	The vaccine was effective, safe, and immunogenic in *H. pylori*-naive children.

In a Phase I trial, an oral inactivated whole-cell *H. pylori* vaccine induced significant lymphoproliferative responses and IFN-γ production in *H. pylori*-negative volunteers. However, the same vaccine failed to eradicate the bacterium in infected individuals (89). Early clinical research also explored vaccines based on *H. pylori* urease combined with the LT adjuvant. In a therapeutic trial involving *H. pylori*-infected adults, a urease–LT vaccine did not confer sterilizing immunity, although it did significantly reduce gastric bacterial density (95). A subsequent study in healthy, *H. pylori*-naive adults confirmed that the same candidate was immunogenic; this trial is classified as not applicable (NA) in **Table 3** because it was not designed to distinguish prophylactic from therapeutic use, its primary objectives being to establish a safe and effective adjuvant dose and to confirm the safety of recombinant urease (96). An alternative approach using rectal delivery of the urease–LT combination failed to elicit any immune response to urease (42).

Recombinant bacterial vector vaccines expressing *H. pylori* urease have also been assessed in human clinical trials. The earliest studies, which employed avirulent *Salmonella enterica* serovar Typhi Ty1033 or Typhimurium as the expression vectors, yielded disappointing results. Among these formulations, only one elicited a urease-specific antibody response, and even this response exhibited considerable inter-individual variability (97, 98). In a subsequent trial, an oral vaccine based on *Salmonella enterica* serovar Typhi Ty21a expressing both urease subunits induced only weak immune responses in *H. pylori*-negative individuals; none of the vaccinated subjects developed a urease-specific serum antibody response, although a subset generated a T cell memory response (99). The same group then examined whether pre-existing immunity to the Ty21a vector influenced the host response to the recombinant vaccine and found that prior exposure to the vector did not alter it (100). Consistent with the earlier findings, a urease-specific humoral response was undetectable in most individuals, whereas a substantial proportion developed a urease-specific T cell memory response.

The Ty21a-based vaccine was further evaluated in a controlled human challenge study in *H. pylori*-negative individuals (101). Participants received multiple doses of either the urease-expressing *Salmonella* strain or a control recombinant strain expressing the *H. pylori* antigen HP0231, and were then challenged with a human *H. pylori* strain previously characterized for use in such studies. Challenge induced the immune responses typically associated with infection, including increased serum cytokine levels and histologic gastritis, together with a rise in urease-specific peripheral blood antibody-secreting cells that had not been detectable beforehand. A reduction in bacterial load occurred in 8 of 33 vaccinated individuals, but a comparable reduction was seen in 5 of 25 control individuals who received the Ty21a vector alone, so no vaccine-attributable protection could be demonstrated. Although these recombinant attenuated vaccines were well tolerated, none conferred protection; efficacy might be improved by incorporating additional *H. pylori* antigens to generate multivalent vaccine strains.

Whereas *Salmonella* strains expressing *H. pylori* urease proved poorly immunogenic in *H. pylori*-negative individuals, a Phase I study of a multivalent vaccine containing CagA, VacA, and NAP induced specific cellular immune responses in *H. pylori*-negative volunteers; in contrast to the vaccines described above, this candidate was administered by the intramuscular (IM) route (102). Building on this work, the investigators introduced a human challenge model in a Phase I/II study in order to obtain a preliminary assessment of protective efficacy, but the multivalent vaccine failed to protect healthy individuals against challenge with a CagA-positive strain (103). A notable exception is the Phase III trial of an oral recombinant vaccine combining UreB with the B subunit of *E. coli* heat-labile toxin (LTB) as a mucosal adjuvant, which proved effective, safe, and immunogenic in *H. pylori*-naive children (94).

A review of the literature over the past five years reveals no new *H. pylori* vaccine candidate that has advanced to a Phase II or Phase III clinical trial with reported outcomes (91). The most definitive findings therefore remain those of the Phase III trial reported by Zeng and colleagues in 2015. This lack of progress reflects the considerable difficulty of developing a vaccine against this pathogen. Basic research remains active, but clinical translation has proven slow. One major challenge is biological. *H. pylori* has co-evolved with humans for tens of thousands of years and has developed sophisticated immune evasion strategies that pose a fundamental barrier to vaccine development (104). An equally important challenge is industrial. Even promising clinical results can be derailed by manufacturing difficulties. The oral recombinant *H. pylori* vaccine developed in China provides a cautionary example. Although it received a Class I New Drug Certificate, commercialization was later abandoned after a plant relocation because of funding shortages and the failure to obtain Good Manufacturing Practice (GMP) certification for the revised production process (104).

Despite these difficulties, immunoinformatics-based multi-epitope vaccines, mRNA-based vaccines and AI-driven target discovery may offer new opportunities for overcoming these obstacles.

## 5 Emerging technologies: rational design of next-generation vaccines

### 5.1 Immunoinformatics-based multi-epitope vaccines

Immunoinformatics, an interdisciplinary field bridging bioinformatics and immunology, has become a cornerstone of modern vaccine design. Through computational modeling and predictive analytics, it improves both the efficiency and the precision of vaccine design. In the design of multi-epitope vaccines against *H. pylori*, immunoinformatics is applied principally to the systematic screening and evaluation of antigenic epitopes (**Figure 3**) (105–107), and it offers distinct advantages over traditional approaches. The pathogenesis of *H. pylori* depends on two categories of factor: adhesion factors such as urease, Lpp20, and the Cag pathogenicity island-associated adhesin L (CagL), which facilitate gastric colonization; and virulence factors such as CagA, VacA, and gamma-glutamyl transferase (GGT), which directly damage gastric mucosal cells (108). Because most of these factors are immunogenic, they are prioritized as epitope sources in vaccine design. Cytotoxic T lymphocyte (CTL) and helper T lymphocyte (HTL) epitopes restricted by major histocompatibility complex (MHC) class I and class II alleles are predicted, and candidate peptides are systematically screened to ensure high binding affinity. The selected epitopes are then assembled into a chimeric construct in which an N-terminal adjuvant and flexible linker sequences are introduced to ensure correct processing and structural stability (109–112). Following *in silico* design, the engineered vaccine molecule undergoes structural verification using a suite of bioinformatics tools. Programs such as ProtParam are used to analyze fundamental physicochemical parameters, including molecular weight, isoelectric point, and stability, while advanced structure prediction tools such as AlphaFold2 generate three-dimensional models that are subsequently validated using ProSA-web for model quality, Ramachandran plots for residue rationality, and ERRAT for structural error assessment, so as to ensure native-like conformations (111, 113, 114). For immunological simulation and cross-reactivity assessment, molecular docking simulations predict the capacity of the vaccine to interact with immune receptors such as MHC molecules and Toll-like receptors (TLRs), and cross-reactivity with human proteins is evaluated in order to minimize the risk of autoimmunity (115, 116). Immunoinformatics therefore provides a robust, efficient, and cost-effective route to *H. pylori* vaccine design and markedly accelerates candidate discovery and preliminary screening; its ultimate success, however, remains contingent on validation and refinement through conventional experimental biology.

**Figure 3:**
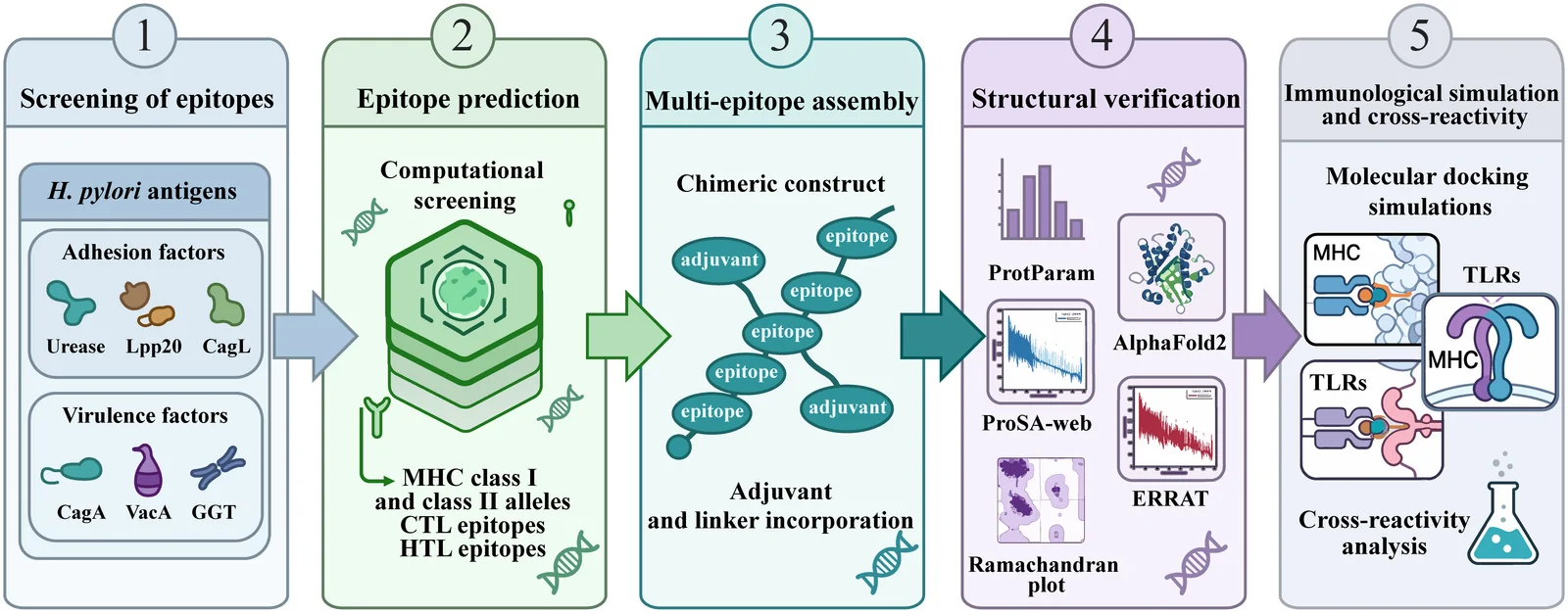
Immunoinformatics workflow for multi-epitope vaccine design against *H. pylori.* The design process follows a five-step computational pipeline. Step 1: Screening of epitopes. Candidate epitopes are derived from *H. pylori* adhesion factors (Urease, Lpp20, CagL) and virulence factors (CagA, VacA, GGT). Step 2: Epitope prediction. CTL and HTL epitopes restricted by MHC class I and II alleles are predicted through computational screening. Step 3: Multi-epitope assembly. Selected epitopes are assembled into a chimeric construct incorporating adjuvant and flexible linker to ensure proper processing and stability. Step 4: Structural verification. The vaccine construct is validated using ProtParam (physicochemical analysis), AlphaFold2 (three-dimensional structure prediction), ProSA-web (model quality), ERRAT (error assessment), and Ramachandran plots (residue rationality). Step 5: Immunological simulation and cross-reactivity. Molecular docking predicts interactions with MHC molecules and TLRs, and cross-reactivity with human proteins is assessed to minimize autoimmune risk. This computational approach accelerates vaccine candidate discovery, though experimental validation remains essential. CTL, cytotoxic T lymphocyte; HTL, helper T lymphocyte; MHC, major histocompatibility complex; TLR_S_, Toll-like receptors; GGT, gamma-glutamyl transferase. Created with Adobe Illustrator.

The *H. pylori* multi-epitope vaccine exemplifies the rational design of a next-generation vaccine using modern bioinformatics. In contrast to conventional vaccines based on whole inactivated or attenuated bacteria, this strategy centers on the systematic screening of immunodominant epitopes drawn from several key virulence proteins (117). The selected fragments are genetically fused through flexible linkers to construct a chimeric vaccine candidate. Commonly targeted *H. pylori* antigens include urease, particularly UreB, the virulence factors CagA and VacA, adhesion factors such as *H. pylori* adhesin A (HpaA), and additional proteins including NAP and Hsp60 (108, 118–120). Among these, UreB is the most frequently used immunogen and has consistently been shown to stimulate a protective immune response against *H. pylori* infection (121, 122), it is therefore commonly prioritized in multi-epitope vaccine design (123). A 2024 study, for example, designed a novel construct incorporating epitopes from nine proteins: UreB, BabA, SabA, VacA, CagA, GGT, high-temperature requirement A (HtrA), NAP, and CagL (106). In another example, the multi-epitope vaccine LL-plSAM-WAE, which contains epitopes from *H. pylori urease*, HpaA, Hsp60, and NAP, induced the secretion of secretory IgA and IgG in mice and significantly reduced gastric bacterial colonization (112).

A recent study illustrates how far this design pipeline has progressed toward experimental validation. By systematically scanning the antigenic landscapes of UreB, flagellin A (FlaA), HpaA, and VacA, the investigators identified and selected epitopes that optimally engage protective CD4^+^ T cell responses and assembled them into a multi-epitope candidate designated UHFV. The construct was displayed on a self-assembling ferritin nanoparticle and formulated with the mucoadhesive polymer hydroxypropyltrimethyl ammonium chloride chitosan in order to improve antigen stability and mucosal residence time. *In vivo* evaluation confirmed that the vaccine elicited potent antigen-specific cellular immunity and substantially reduced gastric *H. pylori* colonization in murine models, and protection was shown to be CD4^+^ T cell-dependent (124). This study is notable because it closes, for a single candidate, the loop from *in silico* epitope selection through nanoparticle presentation and mucoadhesive delivery to demonstrated protection *in vivo*—a sequence that most computationally designed candidates have yet to complete. Because multi-epitope vaccines contain only short, pathogen-specific fragments, however, their intrinsic immunogenicity is often weak. They therefore usually need to be combined with mucosal adjuvants, such as the cholera toxin B subunit (CTB), or with advanced delivery systems, such as lactic acid bacterial surface display and virus-like particles, in order to enhance the immune response and ensure efficacy (125).

Overall, recent research on *H. pylori* multi-epitope vaccines has been characterized by increasingly sophisticated antigen selection and by a diversification of epitope combinations, but key challenges remain in enhancing immunogenicity and optimizing delivery, and these require further investigation.

### 5.2 DNA and mRNA vaccines

Nucleic acid vaccines work differently from protein-based platforms. Rather than delivering the antigen itself, they deliver a DNA plasmid or mRNA encoding an *H. pylori* antigen such as UreB. Once inside host cells, the genetic material is translated into protein, which is then processed and presented to the immune system (126). This approach has several strengths. Because the antigen is produced inside cells, it can stimulate both antibody and cytotoxic T-cell responses—a feature generally considered important for tackling chronic infections (127). Manufacturing is also faster and cheaper than for protein vaccines, since only the gene sequence is needed and there is no requirement to culture live bacteria (128). Another advantage is safety. There is no risk of infection or reversion to virulence, and DNA vaccines in particular are thermostable, so storage and transport are straightforward (129). In addition, a single construct can encode multiple antigens or include cytokine genes such as IL-2 as built-in adjuvants, a level of flexibility not easily achieved with conventional protein vaccines (130).

There are still several obstacles to overcome. Efficient delivery across mucosal surfaces is a major challenge, especially for DNA, standard intramuscular injection rarely generates robust gastric mucosal immunity (131). For DNA vaccines, there is also a theoretical concern about integration into the host genome, though the risk appears very low. Potency is a third issue: plasmid DNA alone often induces weaker antibody responses than protein antigens formulated with potent adjuvants (132), so modifications or co-expressed cytokines are usually needed to boost efficacy. Overall, nucleic acid vaccines are fast to design, easy to manufacture, and broadly applicable. The real challenge, however, is delivery. How do we ensure that a computer-designed sequence is expressed safely and effectively in human cells? For *H. pylori*, that also means getting it to the gastric mucosa. saRNA and circRNA are more recent variants of linear mRNA vaccines. saRNA carries a gene for an RNA-dependent RNA polymerase, which allows the RNA to replicate inside the cell. This reduces the required dose and prolongs expression. The licensed saRNA COVID-19 vaccine (Kostaive) works at 5μg, whereas conventional mRNA vaccines need 30–100μg. With the NovaVec vector, saRNA persists in mice for at least 54 days, compared with 14 days for conventional mRNA. Furthermore, the double-stranded RNA intermediates generated during replication also provide their own adjuvant activity (133). circRNA, by contrast, has a closed loop structure that resists nuclease degradation, triggers less innate immunity, and supports sustained translation (134). circRNA research has so far focused mainly on cancer and viral diseases; no *H. pylori* studies have been reported, probably because manufacturing is difficult and the field is still young (135). Even so, combining circRNA’s advantages with what we know about *H. pylori* could be a promising direction for next-generation mucosal vaccines. Razzak et al. (2025) recently published the first systematic design of an saRNA-based *H. pylori* vaccine, using a multi-epitope construct that targets UreB, BabA, HpaA, CagA, and VacA (117).

Experimental work on saRNA-based *H. pylori* vaccines is still at an early stage. That raises several questions. How stable is the RNA in the gastric environment, and can it be delivered there efficiently? Current models also do not fully capture the complexity of antigen processing or mucosal immune responses *in vivo*. No candidate has yet been tested *in vitro* or *in vivo*, so the findings so far are largely hypothetical (117). A stepwise validation strategy is needed, starting with cell-based assays and moving to animal challenge studies (136). Oral delivery systems such as nanocarriers or engineered bacterial vectors also need to be developed to get past the gastric mucosal barrier.

### 5.3 AI-driven target discovery, validation, and optimization for *H. pylori* vaccine development

AI-driven target discovery and validation are opening new avenues for *H. pylori* vaccine development by accelerating target screening, optimizing antigen design, and precisely modulating immune responses. Current advances are centered on the systematic discovery of novel targets, in-depth optimization of existing targets, and precise regulation of immunogenicity. The integration of AI with immunoinformatics has shifted vaccine target discovery from trial-and-error approaches to systematic high-throughput screening. AI algorithms enable the rapid identification of potential epitopes across multiple clinical strains through large-scale genomic and proteomic mining, and facilitate multi-parameter selection based on antigenicity, conservation, and virulence, thereby expanding the target repertoire from classical antigens such as UreB and CagA to novel candidates including BabA and HpaA (117). In 2025, B-EPIC was introduced as the first B cell epitope prediction model based on the Transformer architecture, achieving area under the curve (AUC) values of 0.882 on an Immune Epitope Database test set (n = 23,888) and 0.945 on a *Trypanosoma cruzi* peptidome dataset (n = 239,575); experimentally, peptides derived from *H. pylori* selected by this model demonstrated effective B cell activation (137). In the same year, Razzak et al. employed an immunoinformatics pipeline to design a saRNA vaccine targeting UreB, BabA, HpaA, CagA, and VacA, with molecular simulations confirming stable binding to the TLR4 complex (117). In process optimization, Tan et al. applied an artificial neural network coupled with a genetic algorithm to optimize the culture medium for recombinant antigen HpaA, achieving a 93.2% increase in yield and an approximately 18% reduction in raw material costs (138). Furthermore, a team from Nanchang University developed an engineered bacterial outer membrane vesicle-based adjuvant platform (ENAP), in which AI-assisted screening identified IL-17A and IFN-γ as the optimal cytokine combination; oral immunization in mouse models elicited antibody responses lasting over 12 weeks and significantly reduced gastric bacterial loads (139).

Nevertheless, several challenges persist in this field. A considerable “dry–wet gap” remains between computationally predicted immunogenicity and actual *in vivo* responses, and no AI-designed *H. pylori* vaccine has yet entered clinical trials. Current models are predominantly trained on linear epitopes and common HLA alleles, limiting their predictive accuracy for conformational epitopes and population-specific HLA types (140). A systematic review encompassing 119 studies highlighted that data silos, algorithmic bias, and insufficient cross-population validation constitute major barriers to the clinical translation of AI-driven vaccine development (140). Future work should focus on several key areas. These include strengthening the link between computational predictions and experimental validation, incorporating antigenic evolution into vaccine design, and establishing standardized guidelines for evaluation and reporting. These steps are essential if we are to move computationally designed candidates closer to clinical testing.

## 6 Enabling technologies: delivery systems and adjuvants

### 6.1 Next-generation delivery systems

The development of effective delivery systems is crucial for the success of *H. pylori* vaccines, because such systems protect antigenic integrity in the harsh gastrointestinal tract and facilitate the activation of specific mucosal immune responses (141). Current research focuses on overcoming the gastric barriers of acidity and proteolysis and on targeting antigens precisely to intestinal or gastric inductive sites. Oral delivery systems, which mimic the natural route of infection and directly activate gut mucosal immunity, have consequently become a major emphasis (142). Recent innovations aim principally to enhance antigen stability in the acidic stomach and to improve targeting efficiency. Solid lipid nanoparticles (SLNs), for example, have been used to bind and encapsulate a DNA vaccine encoding *H. pylori* UreA, forming a nanocomplex termed lipoplex-A; this system incorporates adjuvant lipids such as monophosphoryl lipid A to enhance antigen delivery while allowing the polarization of the immune response to be tuned, for instance toward IgG1 or IgG2c dominance (143). A second approach uses enteric-coated poly (lactic-co-glycolic acid) (PLGA) nanoparticles coated with HP55, a pH-sensitive polymer, which enables the vaccine to withstand gastric acid and to release antigen in the intestine, thereby inducing both humoral and cellular (Th1/Th17) immunity (144, 145). Lactic acid bacteria, which are probiotics generally recognized as safe, provide another promising platform: they are endotoxin-free, have a well-defined genetic background, adhere strongly to mucosal surfaces, and contribute to gut microbiota balance (146). A lactic acid bacteria surface display system (LL-plSAM) displays *H. pylori* antigens directly on the bacterial surface, so that the engineered bacteria are specifically recognized and internalized by M cells on reaching the gut, efficiently initiating an immune response (110). Extending this strategy, the same group displayed an M cell-targeting multi-epitope antigen (SAM-FAdE) on bacterium-like particles (BLPs) prepared from acid-treated *Lactococcus lactis*, achieving a display efficiency of approximately 90%. After oral immunization, the particles targeted M cells of murine Peyer’s patches and promoted antigen transport to dendritic cells, eliciting robust mucosal secretory IgA, CD4^+^ T cell (Th1/Th17), and humoral responses and significantly inhibiting gastric adhesion of *H. pylori* (125). *Bacillus subtilis* spores have likewise attracted attention as oral vaccine vehicles because of their exceptional stress resistance, excellent safety profile, and intrinsic immunogenic properties; *H. pylori* urease antigens (UreA and UreB) have been successfully expressed on the spore surface, and oral administration effectively induced protective immunity (147). Beyond oral delivery, the intranasal route offers an alternative that bypasses the gastrointestinal tract entirely: nanoemulsions prolong antigen retention in the nasal cavity, enhance uptake by dendritic cells, and effectively stimulate a Th1 response (80).

In summary, whether through encapsulation technologies for oral formulations (such as nanoparticles) or alternative administration routes (such as intranasal vaccines), the primary goal is to ensure that vaccine antigens reach their target sites intact (**Figure 4**). Most current research remains in the preclinical stage. Successful clinical translation will require further breakthroughs, including comprehensive assessment of manufacturing process stability, cost-effective scale-up, long-term safety evaluation, and ultimately, validation of efficacy in human trials.

**Figure 4:**
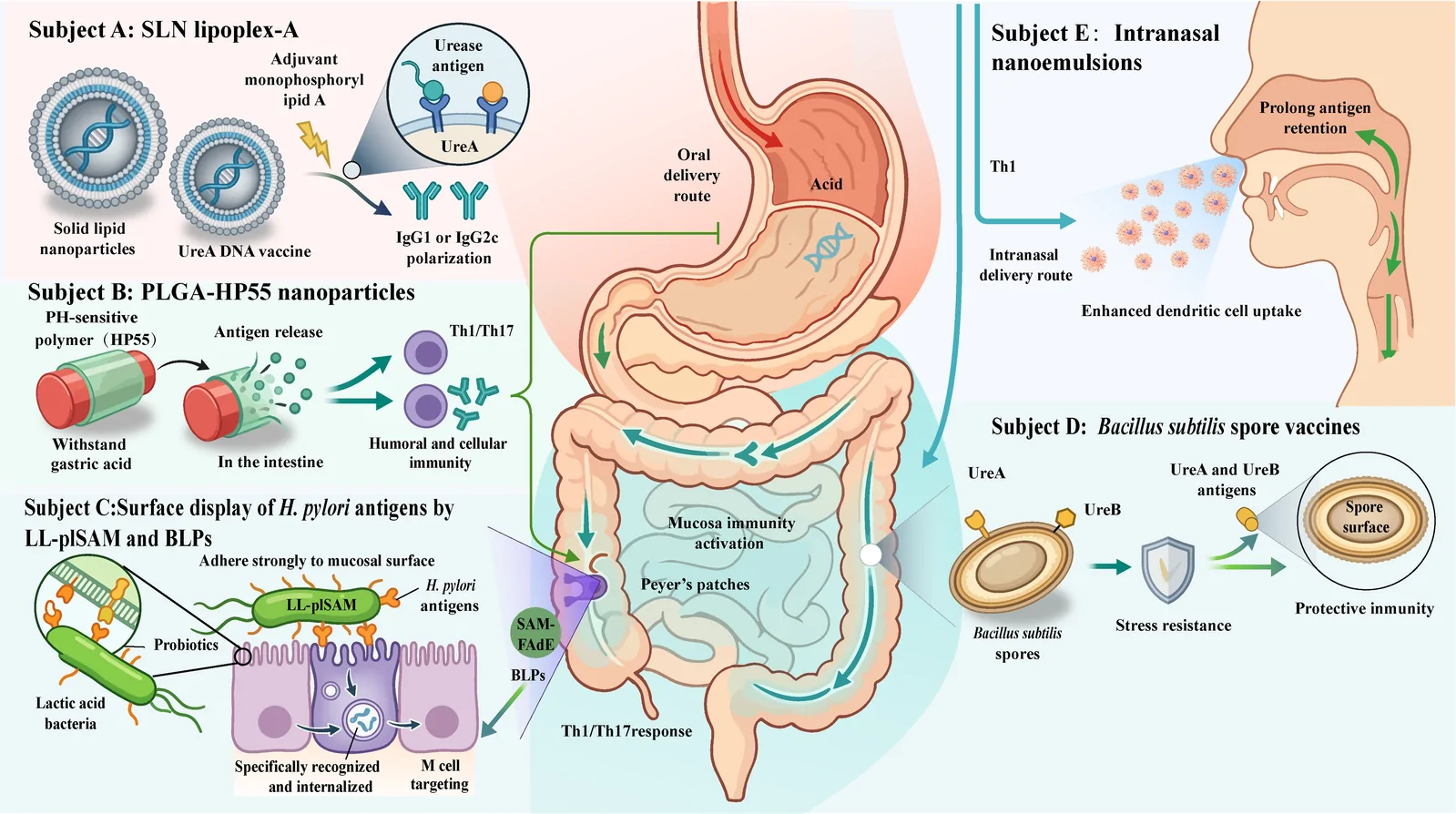
Representative diverse delivery routes for *H. pylori* vaccines. The oral routes (Subject A-D) aim to overcome gastric acid and proteolytic degradation while targeting intestinal immune inductive sites. Subject A utilizes solid lipid nanoparticle (SLN) to form a lipoplex-A complex with a UreA DNA vaccine and monophosphoryl lipid A adjuvant, promoting IgG1 or IgG2c polarization. Subject B employs PLGA nanoparticles coated with the PH-sensitive polymer HP55 to withstand gastric acid and release antigens in the intestine, thereby inducing humoral and cellular (Th1/Th17) immunity. Subject C utilizes probiotics to display *H. pylori* antigens on Lactic acid bacteria (LL-plSAM) and the multi-epitope antigen SAM-FAdE on bacterium-like particles (BLPs). After being specifically recognized and internalized by M cells, these constructs enter Peyer’s patches, and trigger Th1/Th17 immune response. Subject D (*Bacillus subtilis* spore) exploits its exceptional stress resistance to display UreA/UreB on the spore surface, thereby eliciting protective immunity. The intranasal route (Subject E, top right corner) uses nanoemulsions to prolong antigen retention and enhance dendritic cell uptake, effectively stimulating Th1 responses. Collectively, these delivery strategies are designed to preserve antigen integrity and activate specific mucosal immunity. Created with Adobe Illustrator.

### 6.2 Advanced adjuvants for mucosal immunity

A defining characteristic of *H. pylori* is its capacity to survive the extreme acidity of the stomach and to establish long-term colonization through mucosal infection. Given this site of infection, conventional injectable vaccines often fail to induce effective immune protection at the gastric mucosa, and mucosal vaccines have therefore become a primary focus of *H. pylori* vaccine development (148). Adjuvants are crucial for enhancing vaccine immunogenicity and are particularly important for mucosal formulations. An ideal adjuvant for an *H. pylori* vaccine should enhance antigen stability, promote mucosal penetration, and steer the immune response in a protective direction (85, 149). Early studies frequently used bacterial toxin derivatives such as *Escherichia coli* LT and CT; although these adjuvants potentiated immune responses effectively, their toxicity—diarrhea in particular—limited their clinical applicability (150, 151). Subsequent research has therefore concentrated on safer and more effective alternatives. CpG oligonucleotides (CpG-ODN), which contain unmethylated cytosine-phosphate-guanine motifs, represent one such candidate. As a mucosal adjuvant, CpG-ODN activates the Toll-like receptor 9 (TLR9) pathway and stimulates dendritic cell and B cell activation, thereby enhancing immune responses. In mice given *H. pylori* whole-cell sonicate orally with CpG-ODN, mucosal immune responses were comparable to those induced by CT (152). Outer membrane vesicles (OMVs), naturally secreted nanovesicles of 30–250nm released by Gram-negative bacteria, are rich in pathogen-associated molecular patterns and can effectively cross the intestinal epithelial barrier to interact with dendritic cells in the lamina propria (153). A research team at Nanchang University in China genetically engineered *H. pylori* OMVs to synthesize a monophosphoryl lipid A structure with adjuvant activity and to eliminate the antigenic mimicry conferred by *H. pylori* lipopolysaccharide, thereby improving the immunostimulatory capacity of the vesicles. A distinctive advantage of OMVs is their dual function as antigen carrier and adjuvant. Studies in gerbils indicate that *H. pylori*-derived OMVs contain numerous extracellular and cytoplasmic proteins, are multivalent, and induce high levels of secretory IgA, effectively triggering mucosal immunity (154). Combinations of TLR ligands have also shown considerable potential: Poly(I:C) and CpG-ODN, which activate the TLR3 and TLR9 pathways respectively, act synergistically to induce stronger mucosal secretory IgA responses and Th1/Th17-type cellular immunity than either adjuvant alone, and preclinical studies in mice showed that this combination reduced *H. pylori* colonization and alleviated gastritis more effectively (155). In a Phase III clinical trial, the concept of a fusion-based mucosal vaccine was explored by fusing UreB with LTB, generating a recombinant vaccine candidate. In this construct, LTB functions both as a mucosal adjuvant and a delivery vehicle. This design balances adjuvant potency against toxicity: it uses the mucosal targeting property and adjuvant activity of LTB to overcome the tolerogenic barrier of the gastric mucosa, while eliminating the enterotoxic risk associated with the intact toxin. This design enabled safe oral administration and successfully elicited protective immunity in children (94). Lipopeptide adjuvants constitute a further innovative category. Pam2Cys is a prime example, exhibiting self-adjuvanting activity in which the lipid moiety functions as an intrinsic adjuvant and removes the need for an external one. A lipopeptide vaccine was developed by chemically conjugating Pam2Cys to an immunodominant *H. pylori* epitope; the amphiphilic conjugate self-assembles into nanoparticles of approximately 50nm that mimic the size of pathogens and are efficiently taken up by antigen-presenting cells, while Pam2Cys concurrently activates the TLR2/6 pathway to promote dendritic cell maturation and antigen presentation. Intranasal administration of this vaccine induced robust cellular and mucosal immunity and significantly reduced bacterial load in mice (156).

Although several novel adjuvants have shown promising preclinical results, clinical translation remains challenging. A deeper understanding of mucosal immunity, together with new technologies and materials, should nonetheless keep a human *H. pylori* vaccine within reach.

## 7 Current controversies and challenges

To date, no *H. pylori* vaccine has been approved anywhere, and the field has largely remained in preclinical or early clinical development for decades. In our view, a mix of scientific hurdles and open questions accounts for this slow progress, as we outline below.

The first controversy concerns immune escape and antigenic diversity of the pathogen. Natural infection triggers a strong inflammatory response, yet the bacterium persists. This suggests that *H. pylori* actively keeps the host immune system in check. Therefore, a fundamental question arises as to whether a vaccine should aim to prevent colonization or eradicate established infection (87). Most researchers seem to favor prophylactic vaccination as the main public health strategy (157). Either way, a successful vaccine needs to overcome the immunosuppressive environment created by *H. pylori* and elicit protective immunity, particularly Th1 and Th17 responses, that is stronger and more durable than what natural infection provides. Antigenic diversity adds to the difficulty. High-frequency recombination and mutation lead to 20-30% genetic variation between strains, and major virulence factors such as CagA and VacA show strong regional differences. Whether a single antigen like UreB can provide broad protection across different populations remains uncertain, so most researchers now favor multivalent or pangenome-based vaccines. These approaches, however, bring greater formulation complexity and more demanding regulatory pathways (158).

Beyond antigenic diversity, another major obstacle is mucosal immunity and oral delivery. Oral vaccines need to withstand the harsh gastric environment (pH around 1–2) and enzymatic breakdown, but most protein antigens are destroyed before they reach mucosal immune sites such as M cells or gastric-associated lymphoid tissue. Although enteric coatings and nanoparticle carriers have been explored, no stable, clinically useful oral delivery system is yet available (159). The gastric mucosa also lacks the organized antigen-presenting structures found in Peyer’s patches, and existing mucosal adjuvants such as LT and CT derivatives raise safety concerns (160). Consequently, how to durably induce secretory IgA and tissue-resident memory T (TRM) cell responses in the stomach remains unresolved.

Compounding these difficulties is the translational gap between animals and humans.​ Mice and Mongolian gerbils reproduce some features of colonization, but their gastric anatomy and immune responses differ considerably from those of humans. Importantly, these models do not capture the Treg-mediated tolerance that develops over decades of chronic infection in people. This gap probably explains why many candidates that cleared colonization in rodents with over 50% efficacy later failed in Phase I/II trials. In addition, the lack of validated immune correlates of protection in humans means that clinical endpoints and dosing regimens have no solid basis (161).

Economic and regulatory hurdles introduce a different kind of constraint. High-prevalence areas are mostly low- and middle-income countries (162), where cold-chain infrastructure and primary-care vaccination services are often limited (163). Pharmaceutical companies have shown little interest in investing, mainly because pivotal trials would need decades of follow-up with gastric cancer incidence as the final endpoint, because no correlate of protection exists, and because the financial return is uncertain. Moreover, although *H. pylori* is classified as a Group 1 carcinogen by the IARC, no vaccine has been given the same priority as tuberculosis or HPV vaccines, which makes it even harder to secure funding (159).

Given these challenges, current research is moving toward AI-guided identification of conserved, immunodominant epitopes, new mucosal adjuvants, nanoscale delivery systems, and preclinical models that more closely reflect human disease (148). It will take time, but the scientific and public health gains would be well worth the effort.

## 8 Discussion

Chronic *H. pylori* infection remains a major risk factor for gastric adenocarcinoma (164). The organism is usually acquired in childhood and can persist in the human stomach for decades (165), driving progressive inflammation and tissue damage. An effective vaccine is therefore an important public health priority for preventing *H. pylori*-associated gastric cancer. However, no licensed vaccine is yet available for either prophylactic or therapeutic use.

Developing a human *H. pylori* vaccine demands progress on several fronts. Vaccine antigens or epitopes need to elicit strong T cell immunity, not merely antibody responses. A growing body of evidence indicates that CD4^+^ T cells, particularly Th1 and Th17 subsets, are key mediators of protective immunity (55, 64, 65). Another priority is the development of sophisticated delivery systems and potent mucosal adjuvants to generate these protective responses at the gastric mucosa, where the infection takes place. A clearer understanding of the immunological correlates of protection is also needed; reliable biomarkers of vaccine efficacy would help refine formulations and accelerate clinical development. Continued progress in immunoinformatics, particularly AI-assisted epitope prediction and structure-based antigen design, could help address antigenic diversity and identify broadly protective targets. Finally, greater engagement and investment from the pharmaceutical industry are important, because translating promising preclinical candidates into licensed vaccines requires substantial financial and technical resources; without such support, the journey from bench to bedside will remain slow.

Taken together, the evidence reviewed here suggests that the field should rethink its priorities. The human trial record shows that the limiting factor is no longer the availability of immunogenic antigens. UreB, CagA, VacA, NAP, and HpaA all elicit measurable responses in humans, and one UreB-based formulation has already shown Phase III efficacy in children. What we still lack is a strategy to reliably generate the appropriate immune response at the correct anatomical location, and a way to verify that response before committing to large-scale trials. These considerations point to several concrete steps that deserve attention. One clear step is to adopt a standardized panel of immunological endpoints that would be reported uniformly across preclinical and early clinical studies. This panel should include mucosal secretory IgA, gastric IL-17A and IFN-γ, and the frequency of gastric Tregs. Standardization would allow direct comparisons between candidates and help identify correlates of protection from accumulated evidence across multiple trials, rather than relying on isolated studies. Another necessary step is the routine adoption of an iterative approach where computationally designed constructs move through a defined validation pipeline, starting with cellular assays and advancing to animal challenge studies, with experimental results fed back to refine predictive models. The absence of such iteration is the main reason why the growing number of *in silico* vaccine designs has so far generated very little experimental data. A further priority is renewed investment in controlled human infection models. These are ethically and logistically difficult, but they offer the most direct way to test protective efficacy within a reasonable timeframe.

## 9 Conclusion

The development of an *H. pylori* vaccine remains a complex but attainable goal. The field stands at a productive juncture at which advances in mucosal immunology, novel adjuvant technologies, and structure-based vaccine design are converging. The obstacles are formidable, and they are as much translational and economic as they are immunological. Nevertheless, continued scientific and technological innovation, coupled with a deepening understanding of host–pathogen interaction, provides reasonable grounds for anticipating significant breakthroughs in the years ahead.
